# A *Glycine max* sodium/hydrogen exchanger enhances salt tolerance through maintaining higher Na^+^ efflux rate and K^+^/Na^+^ ratio in *Arabidopsis*

**DOI:** 10.1186/s12870-019-2084-4

**Published:** 2019-11-05

**Authors:** Tian-Jie Sun, Long Fan, Jun Yang, Ren-Zhi Cao, Chun-Yan Yang, Jie Zhang, Dong-Mei Wang

**Affiliations:** 1 Hebei Key Laboratory of Plant Physiology and Molecular Pathology, Hebei Agricultural University, Baoding, 071000 Hebei China; 2 Department of Computer Science, Pacific Lutheran University, Tacoma, WA 98447 USA; 3 Hebei Food and Oil Crops Institute, Shijiazhuang, 050031 Hebei China

**Keywords:** Soybean, *GmNHX1*, Salt stress, VIGS, K^+^/Na^+^ ratio

## Abstract

**Background:**

Soybean (*Glycine max* (L.)) is one the most important oil-yielding cash crops. However, the soybean production has been seriously restricted by salinization. It is therefore crucial to identify salt tolerance-related genes and reveal molecular mechanisms underlying salt tolerance in soybean crops. A better understanding of how plants resist salt stress provides insights in improving existing soybean varieties as well as cultivating novel salt tolerant varieties. In this study, the biological function of *GmNHX1*, a NHX-like gene, and the molecular basis underlying *GmNHX1*-mediated salt stress resistance have been revealed.

**Results:**

We found that the transcription level of *GmNHX1* was up-regulated under salt stress condition in soybean, reaching its peak at 24 h after salt treatment. By employing the virus-induced gene silencing technique (VIGS), we also found that soybean plants became more susceptible to salt stress after silencing *GmNHX1* than wild-type and more silenced plants wilted than wild-type under salt treatment. Furthermore, *Arabidopsis thaliana* expressing *GmNHX1* grew taller and generated more rosette leaves under salt stress condition compared to wild-type. Exogenous expression of *GmNHX1* resulted in an increase of Na^+^ transportation to leaves along with a reduction of Na^+^ absorption in roots, and the consequent maintenance of a high K^+^/Na^+^ ratio under salt stress condition. *GmNHX1*-GFP-transformed onion bulb endothelium cells showed fluorescent pattern in which GFP fluorescence signals enriched in vacuolar membranes. Using the non-invasive micro-test technique (NMT), we found that the Na^+^ efflux rate of both wild-type and transformed plants after salt treatment were significantly higher than that of before salt treatment. Additionally, the Na^+^ efflux rate of transformed plants after salt treatment were significantly higher than that of wild-type. Meanwhile, the transcription levels of three osmotic stress-related genes, *SKOR, SOS1 and AKT1* were all up-regulated in *GmNHX1*-expressing plants under salt stress condition.

**Conclusion:**

Vacuolar membrane-localized *GmNHX1* enhances plant salt tolerance through maintaining a high K^+^/Na^+^ ratio along with inducing the expression of *SKOR, SOS1 and AKT1*. Our findings provide molecular insights on the roles of *GmNHX1* and similar sodium/hydrogen exchangers in regulating salt tolerance.

## Background

Plants are subjected to various biotic as well as abiotic stresses during their growth. Due to increasingly exacerbated salinization worldwide [1], compounded with the abiotic stresses such as cold and drought, the damage caused by salt stress has been significantly worse. Plants reserve many ways to tolerate salt stress, including efflux of salt and intracellular partitioning [2]. Salt tolerance, similar to many other metabolic processes, requires the proper control of cellular pH [3]. Na^+^/H^+^ exchangers (NHXs) are integral membrane transporters that catalyze the electroneutral exchange of K^+^ or Na^+^ for H^+^ and are implicated in cell expansion [4], development [5], ion homeostasis [6] and salt tolerance [7]. The *Arabidopsis* genome contains eight NHX homolog-encoding genes which are grouped based on their sequence similarity and localization into three distinct classes, those enriches in plasma membrane (NHX7/SOS1 and NHX8), endosomal/vesicular (NHX5, NHX6), and vacuolar membrane (NHX1, NHX2, NHX3, NHX4), respectively [2]. In *Arabidopsis,* NHXs that localize in vacuolar and plasma membrane are generally considered critical for maintaining Na^+^/K^+^ homeostasis [8]. NHX5 and NHX6, however, function as pH regulators of Golgi, trans-Golgi network, and pre-vacuolar compartments, regulating the sorting of newly synthesized peptides and the direction of Golgi-cargo movement [9].

Despite that *Glycine soja* (wild soybean) is often unsusceptible to salt stress [10], its close relative *Glycine max* (soybean) is typically osmotic sensitive [11]. Thus, the identification and characterization of endogenous genes that are involved in salt tolerance regulation would substantially benefit genetic breeding of soybeans. Overexpression of *GmNAC15*, a member of the NAC transcription factor family in soybean, enhances salt tolerance in soybean hairy roots [12]. Besides, overexpression of *GmSK1*, one of the multi-subunit E3 ligases, enhances tolerance to high salinity and drought stress when transformed into tobacco (*Nicotiana tobacum*) plants [13]. Overexpression of *GmBIN2*, a serine/threonine kinase related to brassinosteroid sensitivity, increases cellular Ca^2+^ content and reduces Na^+^ content, together enhances salt tolerance in transgenic *Arabidopsis* plants [14]. Previous studies in our lab have demonstrated that the overexpression of *GmNHX1* (Gene ID: LOC100816746) is able to complement the defect of the *Saccharomyces cerevisiae ena1–4, nhx1* and *nha1* mutant and reduce the hindering effect of salt stress on cell growth [15]. In this study, we extend our understanding of the function of *GmNHX1* gene and its roles in regulating salt tolerance. We uncover its subcellular localization as well as how its transcription responds to salt stress in soybeans. We further use *Arabidopsis* ecotype Col-0, a model plant allele that has been used in numerous physiology studies, as a host to evaluate the function and mechanism of *GmNHX1* under salt stress condition. We uncover the role of *GmNHX1* by utilizing this model organism, which is to maintain a higher Na^+^ efflux rate and K^+^/Na^+^ ratio under salt stress. Our findings provide important implications for understanding the molecular basis underlying salt tolerance in plants.

## Results

### *GmNHX1* gene is related to salt stress resistance in plants

**Fig. 1:**
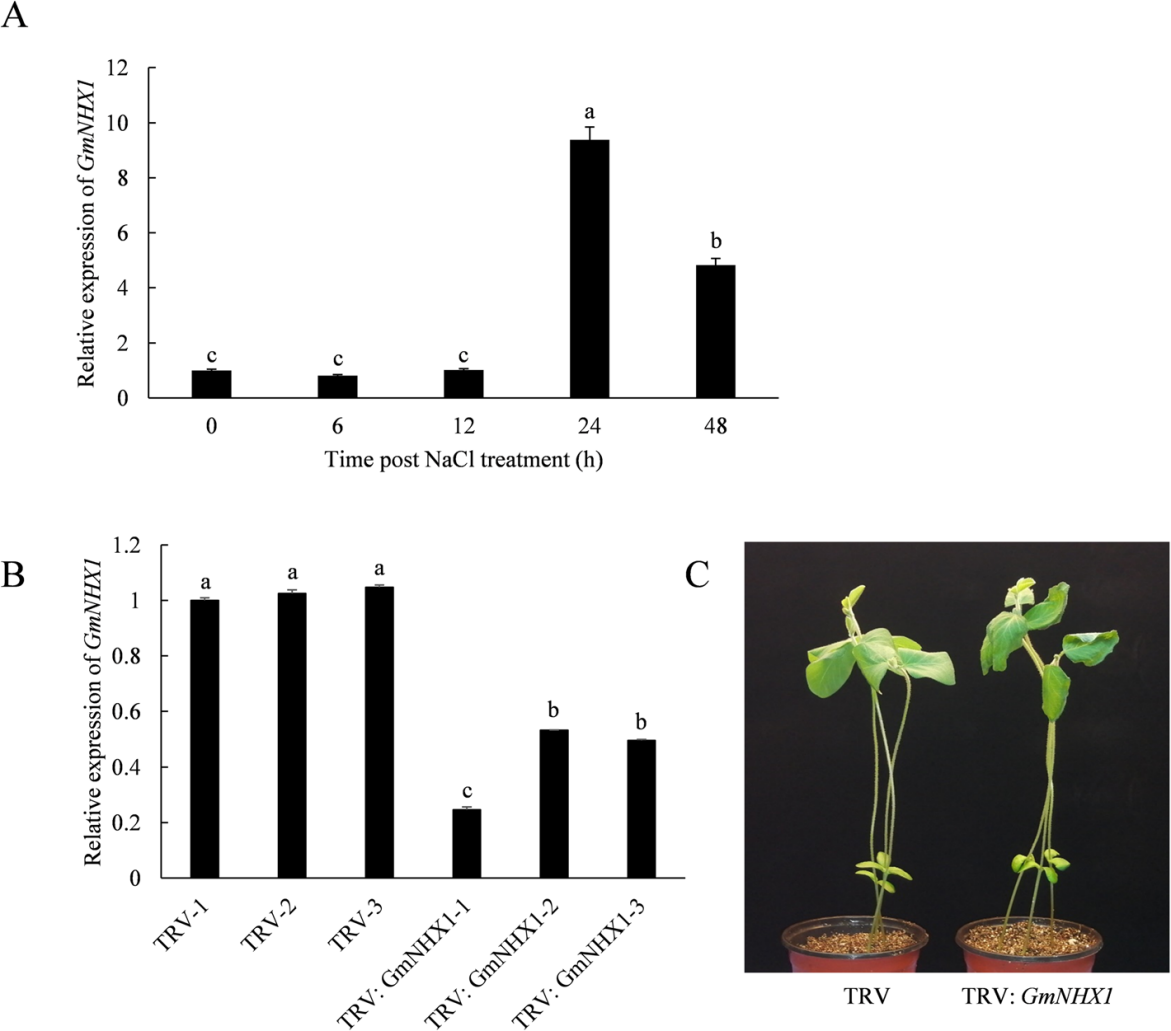
*GmNHX1* positively regulates plant salt tolerance. Transcription Level of *GmNHX1* under salt stress in Soybean was detected by RT-qPCR, *ACTIN* gene was used as a reference gene. The data shows the mean ± S.E. of triplicate experiments. Columns with different letters indicate significant differences at *P* < 0.05 (**a**). The silencing efficiency of *GmNHX1* in silenced plants using VIGS were detected by RT-qPCR, using *ACTIN* as reference gene (**b**). Phenotype of TRV- VIGS plants under 200 mM NaCl solution treatment for 24 h (**c**)

In order to examine the relation between *GmNHX1* and the response to salt stress in soybeans, we examined the transcription level of *GmNHX1* in soybean variety “Jidou-7”. We observed an increase of *GmNHX1* gene expression after salt treatment and the peak of its expression was at 24 h post treatment (hpt), reaching approximately 10-fold of that of before treatment (0 hpt). The expression of *GmNHX1* then began to decrease to approximately 5-fold of that of 0 hpt at 48 hpt (Fig. 1a). The upregulation of *GmNHX1* expression under salt stress implies its relevance to salt stress response in soybeans and its potential role in regulating salt tolerance in soybeans.

We then picked a specific fragment in the *GmNHX1* coding region, to construct the TRV-VIGS vector, and the expression of *GmNHX1* was examined using RT-qPCR after gene silencing. The result showed a satisfactory silencing efficiency after a routine period of TRV-VIGS in soybean plants. Compared to EV (unsilenced plants), TRV vector carrying *GmNHX1* specific fragment reduced the expression level of *GmNHX1* in infected soybeans by nearly 50% (Fig. 1b). As a result of the silencing of *GmNHX1*, the top of the plant drooped and the leaves wilted after 200 mM NaCl solution treatment compared to EV plant (Fig. 1c), suggesting that *GmNHX1* is a critical gene that is involved in the process of plant adaptation to salt stress.

### *GmNHX1* enhances salt tolerance in *Arabidopsis*

**Fig. 2:**
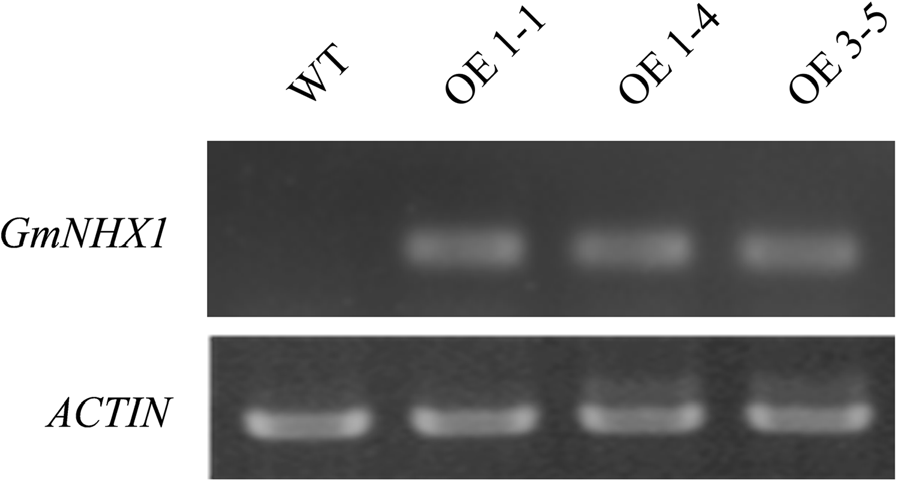
Expression analysis of *GmNHX1* in transformed *Arabidopsis*. Using *ACTIN* as reference gene, RT-PCR was performed to detect the expression of *GmNHX1*

**Fig. 3:**
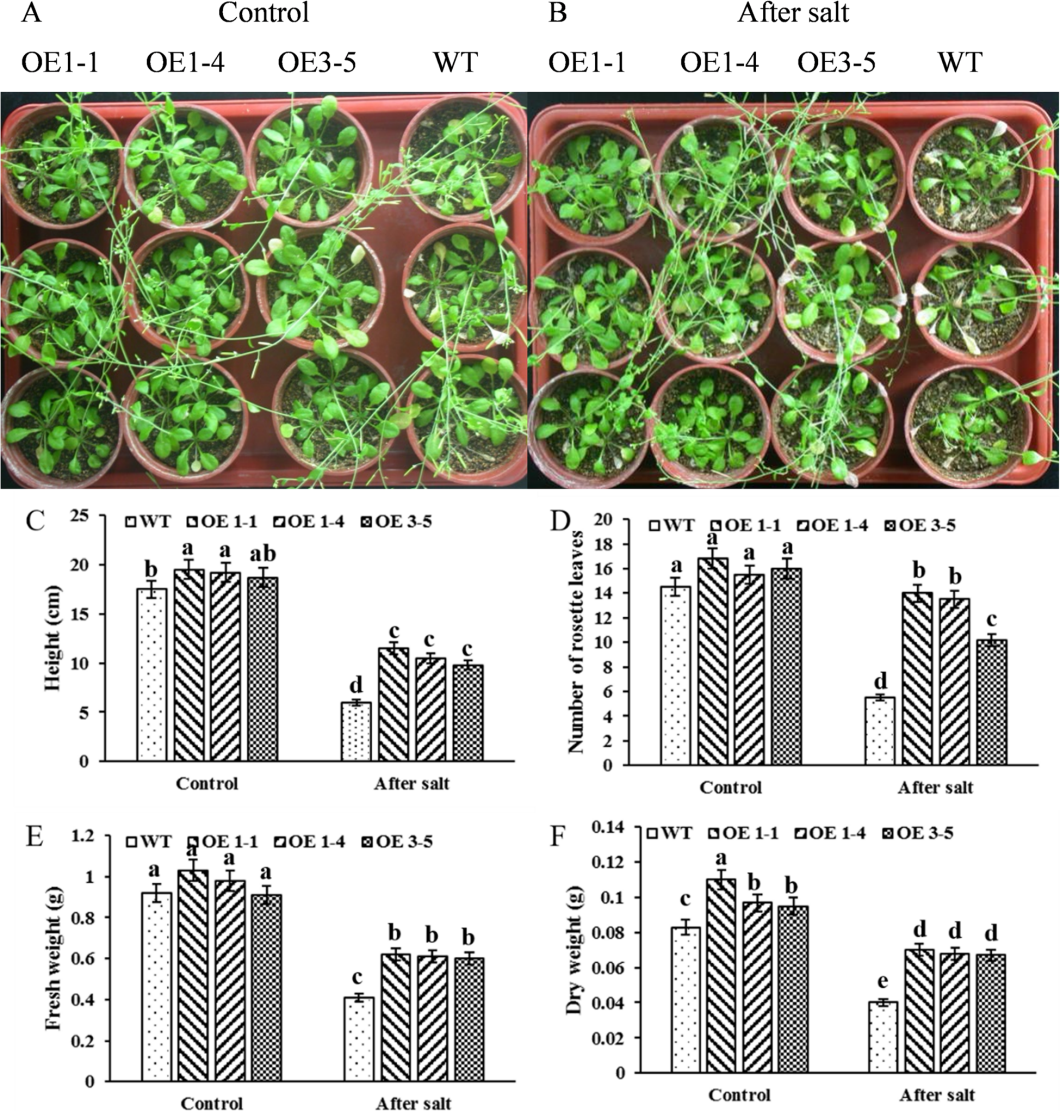
Effect of salt stress treatment on *Arabidopsis* expressing *GmNHX1*. **a, b** *Arabidopsis* phenotype before and after salt treatment; Statistical analysis was performed in measurements of stem length (**c**), number of rosette leaves (**d**), fresh weight (**e**) and dry weight (**f**) before and after salt treatment. The data shows the mean ± S.E. of triplicate experiments. Columns with different letters indicate significant differences at *P* < 0.05

Since the silence of *GmNHX1* reduces salt stress resistance in soybean, we wonder if exogenous expression of this gene could cause the opposite. We constructed the coding sequence of *GmNHX1* gene into a T-DNA within which the *GmNHX1* expression is driven by a *CaMV 35S* promoter, and the construct was then transformed into *Arabidopsis* Col-0. The expression of *GmNHX1* in transformed plants was verified by RT-PCR. We observed significant expression of the gene in transformed plants but undetectable level of the expression in untransformed col-0 (Fig. 2). In order to investigate whether *GmNHX1* overexpression is able to enhance salt tolerance, 21-day-old *Arabidopsis* plants expressing *GmNHX1* were irrigated with 170 mM NaCl solution for 20 days, and plants that were irrigated with water were set as control. Wilting and chlorosis phenotypes were observed in wild-type plants after salt stress treatment, whereas only slight chlorosis was observed in all three lines that express *GmNHX1* exogenously (Fig. 3a, b). Parameters such as stem length, number of rosette leaves, fresh weight, and dry weight were also measured. Plants expressing *GmNHX1* showed significantly higher values in the measurements of stem length (Fig. 3c), rosette leaves (Fig. 3d), fresh weight (Fig. 3e) and dry weight (Fig. 3f), compared to wild-type under salt stress treatment. Taken together, we concluded that *GmNHX1* enhanced plant resistance to salt stress condition.

### GmNHX1 enhances salt tolerance through maintaining K^+^/Na^+^ ratio in root

**Fig. 4:**
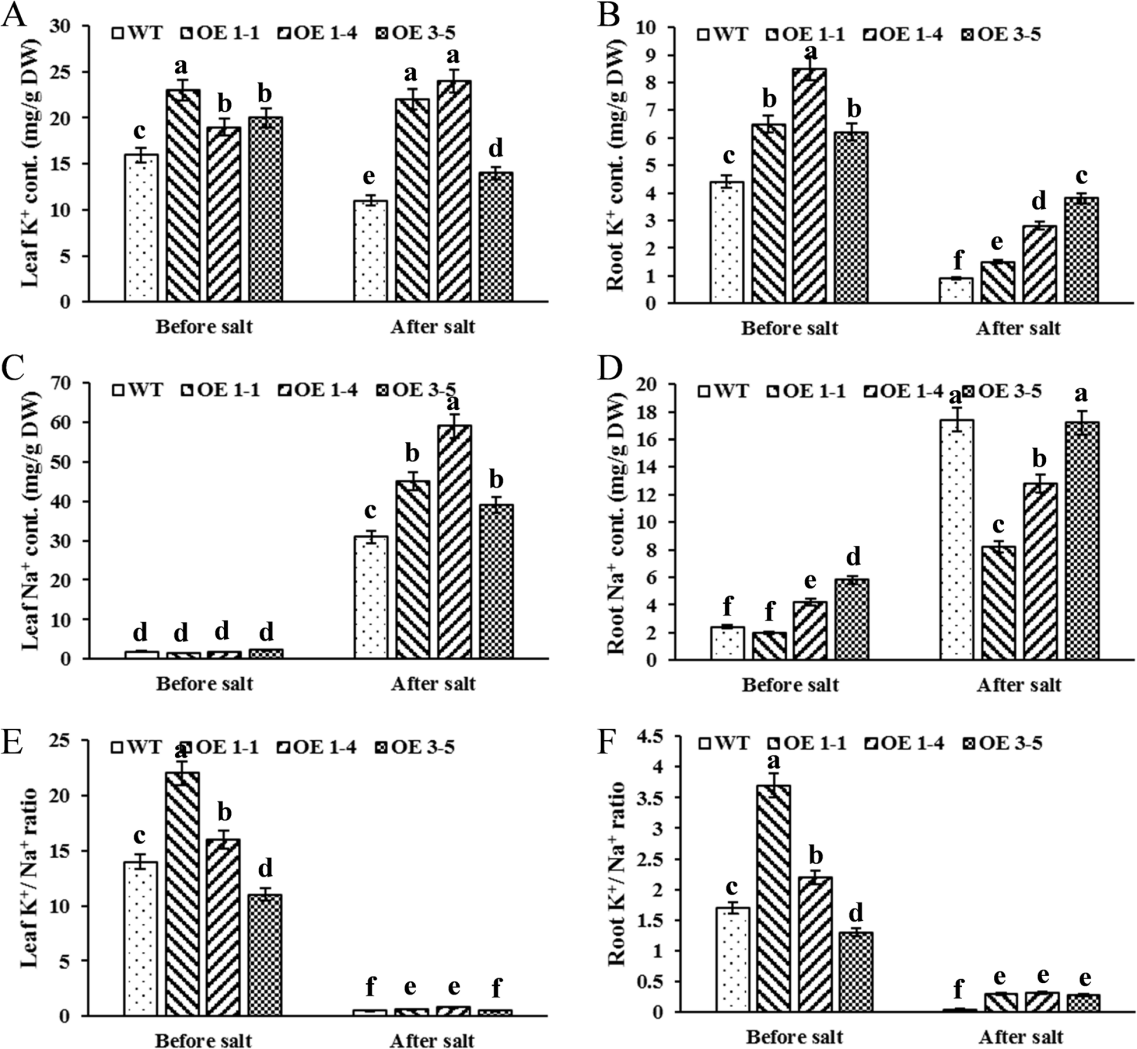
Concentration of Na^+^ and K^+^ in *Arabidopsis* before and after Salt treatment. Total concentration of K^+^ (**a**, **b**) and Na^+^ (**c**, **d**) was measured within the leaf (**a**, **c**) and root tissue (**b**, **d**). Relative K^+^/Na^+^ ratio in the leaf (**e**) and root tissue (**f**) was calculated respectively. The data shows the mean ± S.E. of triplicate experiments. Columns with different letters indicate significant differences at *P* < 0.05

After revealing the positive role of *GmNHX1* in salt resistance, we wondered the molecular mechanism underlying the *GmNHX1*-mediated salt resistance. GmNHX1 has a typical NHX domain, which encodes a functional unit that pumps Na^+^ against its concentration pressure in exchange for a proton. We therefore measured K^+^ and Na^+^ content in *GmNHX1* expressing plants before and after salt stress treatment. K^+^ content in all three *GmNHX1* expressing lines were significantly higher in roots and leaves both before and after salt stress treatment (Fig. 4a, b). As for Na^+^ content, all the three lines expressing *GmNHX1* had no significant difference compared to wild-type plants in leaves before salt stress treatment (Fig. 4c), however all the three lines showed higher Na^+^ level in leaves after salt stress treatment than wild-type (Fig. 4c). In the meantime, Na^+^ content was significantly lower after salt stress treatment in roots of two of the three lines which express *GmNHX1* (OE 1–1 and OE 1–4) compared to wild-type plants (Fig. 4d). These results suggested that exogenous expression of *GmNHX1* might elevate Na^+^ transportation to leaves and reduce Na^+^ content in roots.

The maintenance of a high K^+^/Na^+^ ratio within plant cells is one of the key factors that mediate salt tolerance in plants [16], especially for the root tissue. Compared to K^+^/Na^+^ ratio in leaves (Fig. 4e), K^+^/Na^+^ ratio in roots is significantly higher in all three *GmNHX1* expressing lines compared to wild-type after salt stress treatment (Fig. 4f). GmNHX1 therefore contributes to the high K^+^/Na^+^ ratio in roots, in accordance with our results above.

### Vacuolar membrane-localized GmNHX1 regulates K^+^ and Na^+^ efflux

**Fig. 5:**
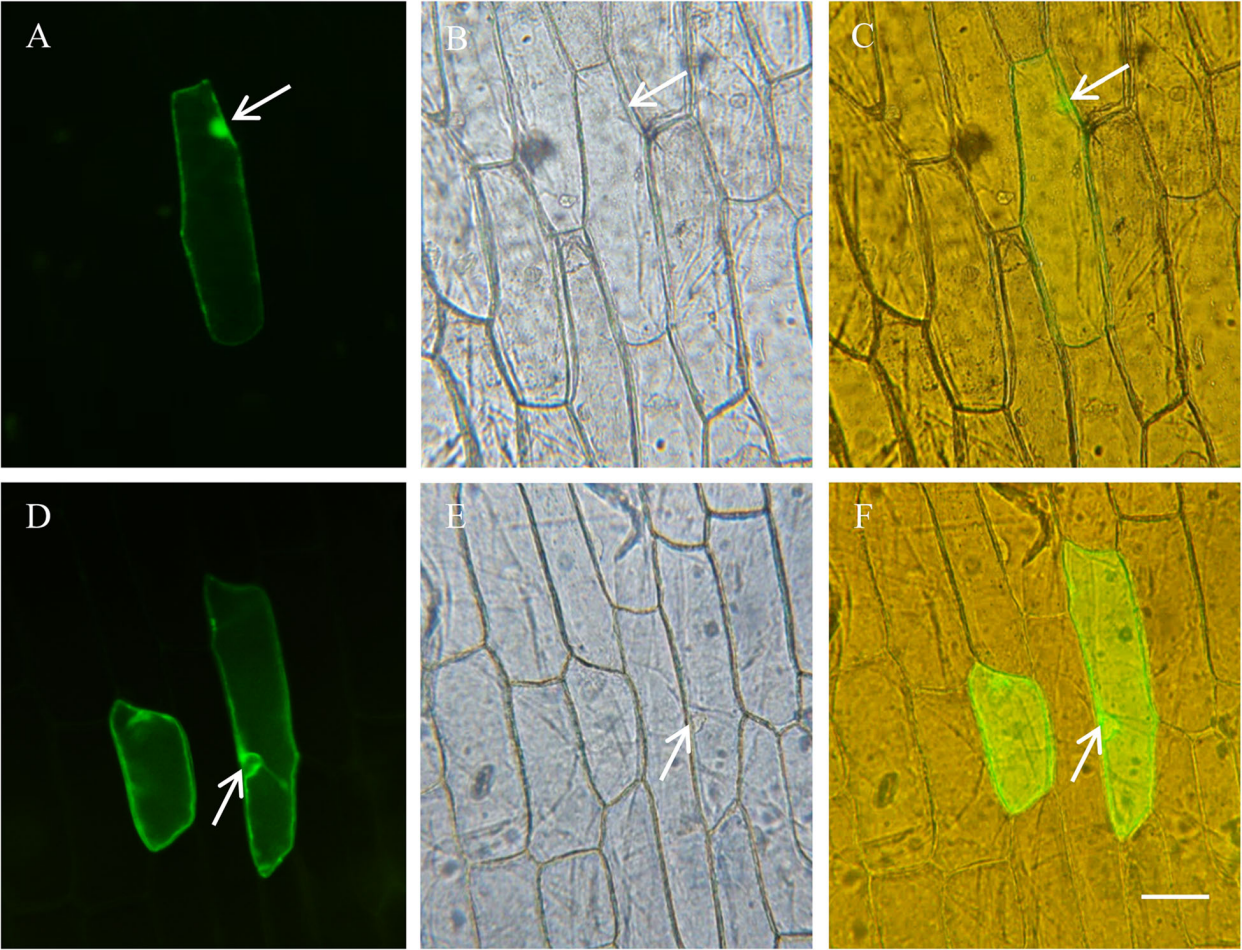
Subcellular localization of GmNHX1. Onion bulb endothelium cells expressing eGFP (**a**-**c**) and *GmNHX1*-eGFP (**d**-**f**) was analyzed under fluorescent microscopy, images were acquired using the 488 nM excitation (**a**, **d**) and light (**b**, **e**). Superimposed images were generated in **c** and **f**, respectively. Arrows indicate the nucleus regions. Bar = 100 μm

**Fig. 6:**
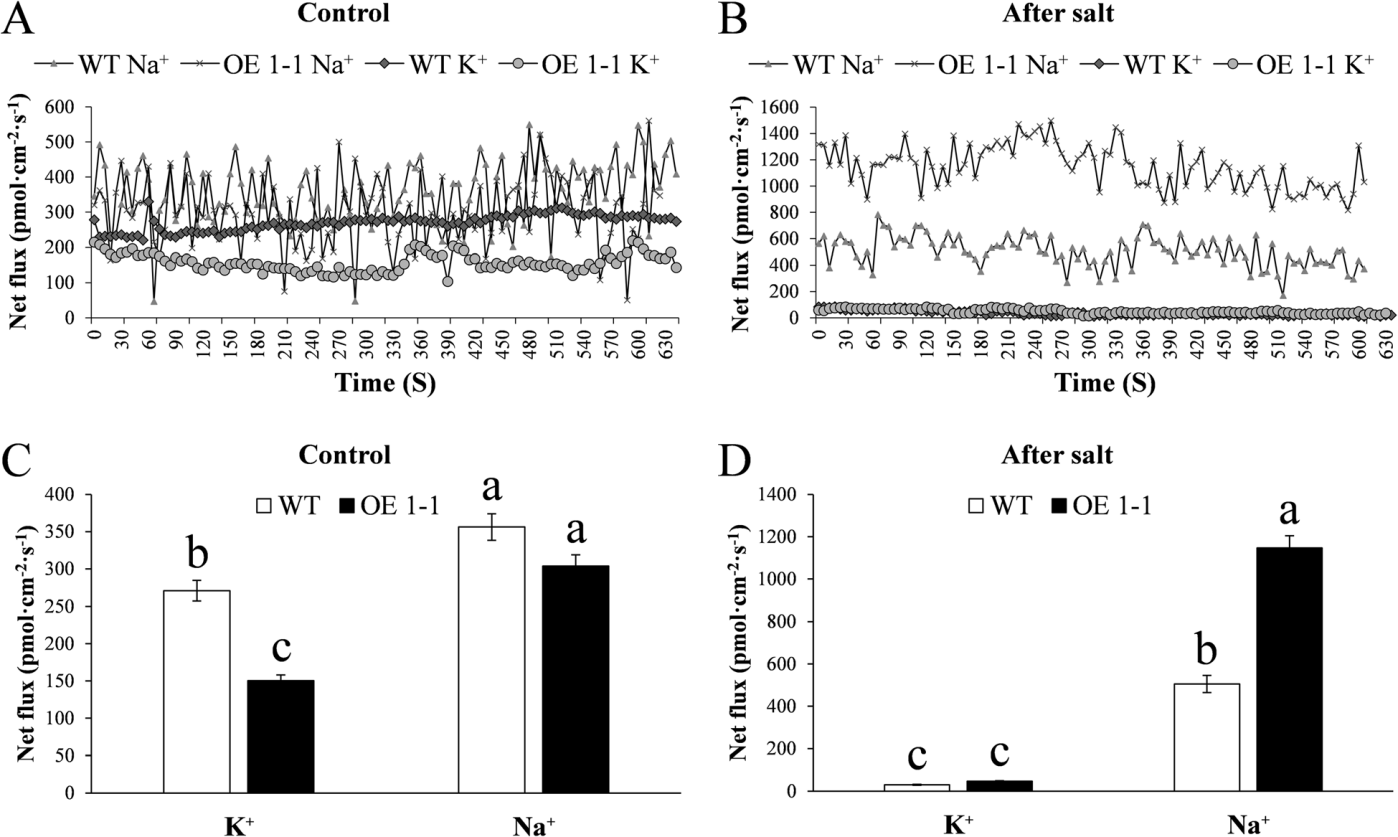
K^+^ and Na^+^ fluxes in root tissues of *Arabidopsis* before and after Salt treatment. Plant materials were subjected to 100 mM NaCl treatment for 24 h. K^+^ and Na^+^ fluxes in roots of wild-type and transformed plants without (**a**) or with (**b**) salt stress treatment are shown. The mean fluxes of K^+^ and Na^+^ without or with salt stress treatment were shown in **c** and **d**, respectively. The data shows the mean ± S.E. of triplicate experiments. Columns with different letters indicate significant differences at *P* < 0.05

Plants overcome salt stress by means of regulating Na^+^ efflux and the partitioning of Na^+^ into vacuolar [2]. Given the observations above, we wondered whether GmNHX1 regulated salt tolerance via adjusting the efflux of these two ion molecules, or modifying the cellular ion storage. To address this question, subcellular localization of GmNHX1 was examined. An eGFP was fused to the C-terminal of GmNHX1 and the resulted fusion protein was constitutively expressed under the control of a *CaMV 35S* promoter. Transformed onion bulb endothelium cells showed a pattern of GFP fluorescence in which signals were enriched in the vacuolar membranes (Fig. 5). To explore the absorption law of Na^+^ and K^+^ under salt stress in *Arabidopsis* alleles expressing *GmNHX1*, we used NMT to detect the flow changes of Na^+^ and K^+^ after 25 h of 100 mM NaCl treatment. The result showed that the efflux of K^+^ in transformed plants were significantly fewer than that of wild-type, with no significant difference for Na^+^ efflux between those before and after salt treatment. The K^+^ efflux of both transformed and wild-type *Arabidopsis* after salt treatment were fewer than that of before salt treatment, but the rate of Na^+^ efflux of transformed plants was greater than that of wild-type. The rate of Na^+^ efflux of both transformed and wild-type *Arabidopsis* after salt treatment was significantly greater than that of before salt treatment, and the rate of Na^+^ efflux of transformed plants after salt treatment was significantly higher than that of wild-type (Fig. 6). This result suggested that *Arabidopsis* plants expressing *GmNHX1*, a vacuolar membrane-localized protein, maintain K^+^/Na^+^ ratio via elevating Na^+^ efflux rate in roots, along with reducing Na^+^ accumulation, which thereby avoiding the toxic effects of excessive salt in cells.

### GmNHX1 regulates stress responsive genes

**Fig. 7:**
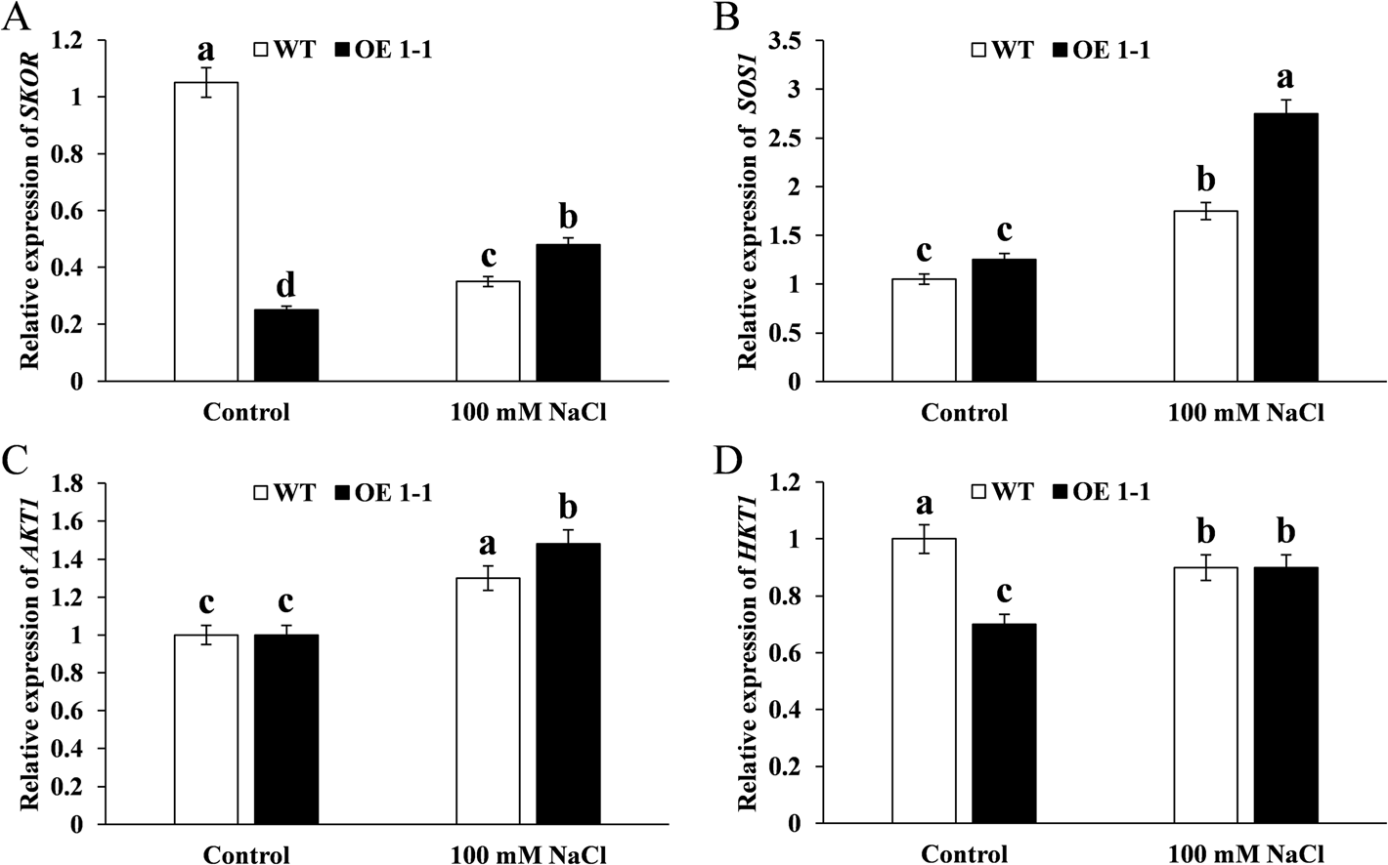
Expression of stress responsive genes in *GmNHX1*-expressing *Arabidopsis*. *Arabidopsis* expressing *GmNHX1* and wild-type plants were treated with 100 mM NaCl for 24 h, relative expression of *SKOR* (**a**), *SOS1* (**b**), *AKT1* (**c**) and *HKT1* (**d**) were detected using RT-qPCR. The data shows the mean ± S.E. of triplicate experiments. Columns with different letters indicate significant differences at *P* < 0.05

The seemingly contradictory observations above, in which GmNHX1 localized in vacuolar membranes whereas it was able to mediate Na^+^ efflux, prompted us to wonder if there were other mechanisms involved. The process of salt stress defense in plants relies on regulation of a series of stress responsive genes [17]. To reveal the relationship between GmNHX1 and stress responsive genes, we employed RT-qPCR to quantify the expression of *AKT1*, *HKT1*, *SOS1* and *SKOR*, which are closely related to salt stress response [2]. Before salt stress treatment, the expression of *SKOR* and *HKT1* in transformed plants were significantly lower than those of wild-type, whereas *SOS1*, *AKT1* showed no significant difference between the two (Fig. 7). Salt stress treatment elevated the expression of *SOS1* and *AKT1*, and reduced the expression of *SKOR* and *HKT1*. After salt treatment, GmNHX1 upregulated the expression of *SKOR, SOS1* and *AKT1*, but not *HKT1*.

## Discussion

Na^+^/H^+^ exchangers (NHX) function as regulators of intracellular ion homeostasis, mainly by increasing Na^+^ excretion of cells (such as SOS1) [18] or compartmenting Na^+^ into vacuolar, such as AtNHX1, to improve plant salt tolerance [19]. Apse et al. suggested that AtNHX1 is critical to the resistance to salt stress in plants [20]. Since *GmNHX1* possesses classic sodium/hydrogen exchanger (NHX) features in its sequence, we therefore wonder if *GmNHX1* is also related to salt stress resistance. In this work, we use VIGS to investigate the function of GmNHX1 in soybeans under salt stress condition. VIGS is a fast, simple and reliable approach that has been used in many functional biology studies, yet is still limited due to the difficulty in finding compatible plant virus stains [21]. TRV (*Tobacco rattle virus*)-mediated VIGS has been widely used in many functional biology studies [22], and is applicable in soybeans according to Liu et al. [23]. We found that the salt resistance has been reduced in *GmNHX1*-silenced plants, suggesting that this gene is closely related to plant salt stress resistance. Previous studies suggest that heterologous expression of chrysanthemum *DgNHX1* is able to improve salt tolerance in tobacco, causing an increase of Na^+^ and K^+^ accumulation in tobacco leaves [24]. ZxNHX1 and ZxVP1–1 could increase the salt and drought resistance of roots, as well as increase the accumulation of Na^+^, K^+^ and Ca^2+^ in leaves [25]. Similarly, Heterologous expression of salt-tolerant plant hippocampus *SbNHX1* can improve salt tolerance of *Jatropha curcas*, increase Na^+^ content and decrease K^+^ content in leaves when exposed to 200 mM NaCl [26]. *Arachis hypogaea* plants expressing *AtNHX1* are resistant to drought, and the content of Na^+^ and K^+^ in leaves are increased [27]. Stress-inducible expression of *TaNHX2* significantly improves growth performance as well as Na^+^ and K^+^ content from the leaf and root tissue of T_2_ transgenic eggplants (*Solanum melongena* L.) under salt stress, compared to non-transformed plants [28]. NHX in sweet sorghum is mainly involved in the transportation of Na^+^, facilitating Na^+^ homeostasis in response to the increase of salt concentration [29]. Our work showed that the contents of Na^+^ and K^+^ in roots have been significantly increased and the K^+^/Na^+^ ratio also increases significantly in plants expressing *GmNHX1* under salt stress, in accordance with these reports.

Previous studies have shown that SOS1, membrane Na^+^/H^+^ exchanger protein, mediates the efflux of Na^+^ in roots [30], previous research shown that the high efficiency K^+^ channel protein HKT1 is located in the membrane, which plays an important role in maintaining the homeostasis of K^+^ and Na^+^ on the aboveground part of the plant [31–33]. SOS1 and HKT1 are located in the membrane, which play key role in regulating K^+^ absorbance from soil to the root cells, and the K^+^ and Na^+^ homeostasis [34]. *AKT1* encodes an internal rectifier K^+^ channel protein, mainly regulates internal K^+^ flow into the root cells [35]. SKOR as an external rectifier K^+^ channel protein mainly involved in K^+^ loading from the column cell to the xylem [36]. Yuan et al. [37] prove that the ZxNHX1 regulates the whole plant K^+^/ Na^+^ homeostasis, and the expression of ion transport protein genes such as *SKOR*, *SOS1*, *AKT1* and *HKT1* were significantly down-regulated. In the NMT result, the net flux rate of K^+^ in transformed plants under normal conditions is significantly lower than that in the WT plants, consistent with the RT-qPCR results that *GmNHX1* down-regulated the expression of *SKOR* and *HKT1* genes in *Arabidopsis* under normal conditions, but had little effect on *SOS1* and *AKT1*. Salt stress induced the expression of *SOS1* and *AKT1*, and decreased the expression of *SKOR* and *HKT1*. Under salt stress, compared with WT, *SKOR*, *SOS1* and *AKT1* in *GmNHX1* transformed plants increased significantly, but not *HKT1*, thich may explain why efflux of Na^+^ in the transformed plants after salt treatment is significantly greater than that in the WT plants.

We show that *GmNHX1* overexpression enhances salt tolerance of *Arabidopsis*. We speculate that, on one hand, by increasing the efflux of Na^+^ in root cells, Na^+^ content is consequently reduced in roots and the K^+^/Na^+^ ratio increases; on the other hand, the Na^+^ is transported to leaves through the xylem, accumulating in the vacuolar in leaves, and K^+^/Na^+^ ratio is maintained stable. When under salt stress, the efflux rate of Na^+^ in roots is greatly increased, and the possible reason is that the expression of intermembrane Na^+^/H^+^ exchanger *SOS1* is induced by GmNHX1. Our results further show that the salt tolerance of the transgenic *Arabidopsis* is mainly achieved by regulating the Na^+^ distribution in plants.

## Conclusions

In summary, soybean Na^+^/H^+^ exchanger *GmNHX1* responds to and regulates plant tolerance to salt stress. In transformed *Arabidopsis* which expresses *GmNHX1*, GmNHX1 changes the flow rate of K^+^ and Na^+^ in root cells by altering the expression of *SKOR* and *SOS1*, in order to regulate the accumulation of K^+^ and Na^+^ in roots and leaves, as well as the maintenance of a high K^+^ /Na^+^ ratio in roots, together improve the tolerance to salt stress in plants.

## Methods

### Cultivation and salt treatment of plant materials

Soybean cv. Jidou-7 was obtained from the Institute of Grain and Oil Crops, Hebei Academy of Agricultural and Forestry Sciences, and was cultivated in a greenhouse with a 14 h light/10 h dark cycle at a constant temperature of 25 °C and 700 μmol photons m^− 2^ s^− 1^. The 10-day-old soybean seedlings were transferred to Hoagland nutrient solution for 24 h, then transferred to Hoagland nutrient solution containing 170 mM NaCl, and sampled at 0 h, 6 h, 12 h, 24 h and 48 h, before RT-qPCR analysis. *Arabidopsis* ecotype Col-0 was obtained from the Arabidopsis Biological Resource Center (ABRC; http://abrc.osu.edu). The wild-type and transformed *Arabidopsis* seeds were surface sterilized and planted on MS medium, then transferred to vermiculite for 20 days and watered in Hoagland nutrient solution. For salt treatment, 21-day-old *Arabidopsis* plants were irrigated with 170 mM NaCl solution, biomass measurement including plant height, rosette number, fresh weight and dry weight, measurement of K^+^ and Na^+^ content and expression quantitation of salt stress related genes were performed 20 days after salt stress treatment initiation.

### Virus induced gene silence

A specific fragment of *GmNHX1* was amplified using primer pair *GmNHX1*-F and *GmNHX1*-R (Table 1), with PrimeSTAR HS DNA Polymerase (TaKaRa). The virus vector that is used to silence *GmNHX1*, pTRV2-*GmNHX1*, was constructed by inserting the amplified fragment of *GmNHX1* into pTRV2 vector between *Bam*H I and *Kpn* I recognition sites. TRV-VIGS was performed according to the previous report [22]. After infection, soybean seedlings were treated with 170 mM NaCl solution for 24 h, then *GmNHX1* silencing efficiency was determined by RT-qPCR.

**Table 1:** Synthetic DNA oligo used in this research

Oligo name	Sequence (5`- 3`)	Application
GmNHX1-F	acgttgcacgggatcccccttcatgccatgggaca	Construction of TRV induced *GmNHX1* silencing vector.
GmNHX1-R	ctagctagggggtacctccagaggaccaacatccaac	
RT GmNHX1 F	actgcgaagcaatgcaatca	Detection of transcriptional level of *GmNHX1* and using RT-PCR.
RT GmNHX1 R	ggccattacgttcagttggtg	
RT ACTIN F	atggctgatggtgaagacattc	
RT ACTIN R	tccatgctcaatagggtacttg	
OE GmNHX1 F	ggtaccatggtttttgaaatcagttc	Construction of binary vector pCAMBIA1300-GmNHX1.
OE GmNHX1 R	tctagatcaacgccattgatggcca	
GFP GmNHX1 F	tgcccatgggacaaaatggtttttgaaatc	Construction of GFP fused vector pCAMBIA1300-GmNHX1-GFP.
GFP GmNHX1 R	cgccccgggacgccattgatgg	
qRT AtSKOR F	accgaaacaaactcggtaggaa	Detection of transcriptional level of salt stress related genes using RT-qPCR.
qRT AtSKOR R	ttagcacggatagagacaggaatg	
qRT AtSOS1 F	gtgaagcaatcaagcggaaa	
qRT AtSOS1 R	tgcgaagaaggcgtagaaca	
qRT AtHKT1 F	gatttgtccccacgaatgaga	
qRT AtHKT1 R	caaaaccaagaagcaagggaac	
qRT AtAKT1 F	aaaggtctcactcatcaacaacga	
qRT AtAKT1 R	tcggcaaaagaggcaaaataag	
qRT ACTIN F	gcaccgccagagagaaaatac	
qRT ACTIN R	caccaccacgaaccagataaga	

### RT-PCR and RT-qPCR

Total RNA was isolated from plant material using UNlQ-10 Column Trizol Total RNA Isolation Kit (Sangon), and reverse transcript with PrimeScript RT reagent Kit with gDNA Eraser (TaKaRa). RT-PCR and RT-qPCR was performed using Ex Taq DNA polymerase (TaKaRa) and SYBR Premix DimerEraser (TaKaRa) according to user manual, respectively, using *ACTIN* as reference gene. Primers used in RT-PCR and RT-qPCR experiments have been listed in Table 1.

### *Arabidopsis* transformation

Full length CDS of *GmNHX1* was PCR amplified using primer pair OE GmNHX1 F/ R (Table 1), and constructed into binary vector pCAMBIA1300 between the restriction enzyme recognize site of *Kpn* I and *Xba* I, under control of *CaMV 35S* promoter.

### In vivo measurement of K^+^ and Na^+^

Dried plant materials were ground into fine powder. Concentrated sulfuric acid was added to the ground powder, and the mixture was boiled at 170 °C for 20 min. A few drops of 30% hydrogen peroxide were added till a large amount of white smoke appeared, followed by digestion at 220 °C for 40 min, then 330 °C for 2 h. The content of Na^+^ and K^+^ ions were measured using a flame spectrophotometer (Sherwood M410).

### Subcellular localization of GmNHX1

Full-length CDS of GmNHX1 was PCR amplified using primer pair GFP GmNHX1 F/ R (Table 1), and constructed into pCAMBIA1300-*GFP*, between the restriction enzyme recognize sites *Nco* I and *Sma* I, and was fused to the N` terminal of GFP, resulting a fusion protein that is expressed under the control of *CaMV 35S* promoter. Purified pCAMBIA1300-*GmNHX1*-*GFP* plasmid was bombarded with a particle gun (BioRad PDS- 1000/He). Transformed onion bulb endothelium cells were cultivated in 1/2 MS medium for 24 h, before analyzed under the fluorescence microscopy (Olympus BX53).

### Measurement of Na^+^ and K^+^ flow rate

Fluxes of Na^+^ and K^+^ ion was measured using NMT. 7-day-old *Arabidopsis* seedlings were transferred to MS medium containing 100 mM NaCl, NMT test was performed by Xuyue (Beijing) Sci.& tech. co., ltd., in accordance with previous report [38].

## Data Availability

The datasets used and/or analyzed during the current study are available from the corresponding author on reasonable request.

## Abbreviations

hpt: Hours post treatment
NHX: Na^+^/H^+^ exchanger
NMT: Non-invasive micro-test technique
PCR: Polymerase chain reaction
TRV: *Tobacco rattle virus*
VIGS: Virus-induced gene silencing
